# Could the erythrocyte indices or serum ferritin predict the therapeutic response to a trial with oral iron during pregnancy? Results from the Accuracy study for Maternal Anaemia diagnosis (AMA)

**DOI:** 10.1186/s12884-016-1005-x

**Published:** 2016-08-12

**Authors:** Cristiane Campello Bresani Salvi, Maria Cynthia Braga, José Natal Figueirôa, Malaquias Batista Filho

**Affiliations:** 1 Nutrition Research Group at Instituto de Medicina Integral Prof Fernando Figueira – IMIP, Rua dos Coelhos, 300, Boa Vista, Recife, PE CEP: 50.070-550 Brazil; 2 Instituto Nacional do Seguro Social/Ministério da Previdência Social – INSS/MPS, Av Jorn Mário Melo, 343, Santo Amaro, Recife, PE CEP: 50.040-010 Brazil; 3 Postgraduate Program in Maternal and Child Health of IMIP, Rua dos Coelhos, 300, Boa Vista, Recife, PE CEP: 50.070-550 Brazil; 4 Postgraduate Program in Public Health at Centro de Pesquisas Aggeu Magalhães – Fundação Oswaldo Cruz – CPQAM/FIOCRUZ, Av. Professor Moraes Rego, s/n - Campus da UFPE - Cidade Universitária, Recife, PE CEP: 50.670-420 Brazil

**Keywords:** Anaemia, Iron-Deficiency, Pregnancy, Erythrocyte Indices, Sensitivity and Specificity, Ferrous Sulfate

## Abstract

**Background:**

Treatment of maternal iron-deficiency anaemia can reduce risks of prematurity and low birth weight; hence a reliable diagnosis of maternal iron needs is critical. However, erythrocyte indices and serum ferritin have shown a weak correlation with iron *status* during pregnancy. This study verified the accuracy of those tests to predict the responsiveness to a *therapeutic test with oral iron* as reference standard for iron deficiency in pregnant women.

**Methods:**

A prospective diagnostic study phase 3 was conducted in a single prenatal care center in Northeast Brazil. Between August 2011 and October 2012 a consecutive sampling included 187 women in their 2^nd^-3^rd^ trimesters of low-risk pregnancy and having anaemia (haemoglobin <11.0 g/dL). Until December 2012, 139 women completed a trial with daily pills of ferrous sulfate (40 mg of iron), during 23 to 125 days. Haemoglobin (Hb), other erythrocyte indices and ferritin (index-tests) were assessed pre-treatment by automated analyzers. Hb was performed also post-treatment to assess the therapeutic response by its post-pretreatment differences. We estimated sensitivity (Se), specificity (Sp), predictive values (PV), likelihood ratios (LR), diagnostic Odds Ratio (OR), area under Receiver Operating Characteristic curve (AUC), accuracy ratio and agreement coefficient of the index-tests against an increase of at least 0.55 Hb Z-score (reference standard test). We calculated the Z-scores according to the reference population from Centers for Disease Control and Prevention.

**Results:**

Hb had a mean increase of 0.24 Z-score after 30 iron pills (*p* 0.013). All index-tests demonstrated PV- above 70 %, PV+ around 40 %, LR around 1.0, and AUC of 0.5 to 0.6. Hb and haematocrit had Se of 50 % (95 % CI 40 to 70); and Sp of 59 % (95 % CI 43 to 74) and 47 % (95 % CI 38 to 57), respectively. Ferritin, Mean Corpuscular Volume, Mean Corpuscular Haemoglobin, Mean Corpuscular Haemoglobin Concentration and Red blood cell Distribution Width had Se below 40 % with Sp above 70 %.

**Conclusions:**

Erythrocyte indices and ferritin could not predict the iron needs of anemic pregnant women. Increases of Hb Z-scores after a short treatment with oral iron may be a reliable therapeutic test.

**Trial registration:**

World Health Organization International Clinical Trials Registry Platform U1111-1123-2605; Brazilian Registry of Clinical Trials RBR-237wbg, registered 28 July 2011

**Electronic supplementary material:**

The online version of this article (doi:10.1186/s12884-016-1005-x) contains supplementary material, which is available to authorized users.

## Background

A reliable diagnosis of maternal iron-deficiency is a critical issue, since an effective therapy with oral iron during pregnancy can prevent perinatal adverse outcomes, such as low birth weight [1, 2] and prematurity [2]. The presence of anaemia, in other words, low levels of haemoglobin concentration (Hb) or haematocrit (HTC), has been considered as a *proxy* for iron-deficiency and widely used to estimate its prevalence and to indicate and evaluate the iron-therapy during pregnancy [3, 4], especially in developing countries [4, 5]. However, maternal anaemia may also be associated with infectious or nutritional diseases and gestational haemodilution [4], as well as the iron-deficiency may be present with normal levels of Hb and HTC [3, 6]. Serum ferritin is the most recommended iron *status* biomarker [3, 4], but it has an iron-storage role rather than functional [6, 7]. Actually, there is a lack of standardized and validated diagnostic screenings to select pregnant women for iron-therapy in clinical trials [8], and so it allows one to question whether iron needs are being correctly identified and treated during pregnancy.

Clinical studies have showed a low accuracy of these tests to diagnose maternal iron-deficiency [9–11]. Hb and HTC [9, 12–14], as well as the other erythrocyte indices [9, 13], do not correlate with the iron *status* in pregnant women, probably due to the physiological haemodilution [15, 16] and ‘emergency hemopoiesis’ [17, 18], which not only modify the cell indices in the erythrogram, but also the serum ferritin and other biomarkers of the body iron [6, 7]. The cutoff points of erythrocyte indices and serum ferritin were based on the distribution curves of populations from developed countries in the 70’s and 80’s [19, 20]; therefore, they were not defined by diagnostic validation studies. Pregnancy validation studies, which compared erythrocyte indices with the serum ferritin [9, 10, 14, 21, 22] or the iron content in the bone marrow [11, 12] describe very low sensitivity (Se) and/or specificity (Sp) for the erythrocyte mass indices (Hb and HTC) [10, 14, 21, 22] and morphology indices (mean corpuscular volume - MCV, mean corpuscular haemoglobin - MCH, mean corpuscular haemoglobin concentration - MCHC, red blood cell distribution width - RDW) [9, 11, 12, 22]. On the other hand, only two studies compared the ferritin levels with the iron in pregnant women’s bone marrow [11, 12], and there is no consensus about its cutoff during pregnancy [7, 23–25].

Thus, a large proportion of pregnant women is susceptible to diagnostic misclassification and decision-making mistakes when the indication of iron-therapy is based on the erythrogram or even ferritin results. Studies which validate those tests against therapeutic outcomes of iron supplementation at a real clinical setting might support this assertion. Therefore, our study assessed the diagnostic performance of erythrocyte indices and serum ferritin to predict and discriminate the increase of Hb in response to a *therapeutic test with oral iron*, which was applied as the reference standard of iron-deficiency in anaemic pregnant women at a prenatal care center. The response to iron-therapy may be a suitable diagnostic alternative, as is being recommended by World Health Organization (WHO) [3] and British Committee for Standards in Haematology [25]. To normalize the effect of haemodilution phenomenon on Hb changes [26], we adjusted the Hb values for the gestational age at start and end of iron-therapy, and we are introducing a *Nomogram of Hb Z-scores* as an easy-to-use graphic to follow the Hb values during pre-natal care.

## Methods

This is a prospective validation study on phase III [27], whose index-tests were the pre-treatment values of serum ferritin, red blood cell count, Hb, HTC, MCV, MCH, MCHC, RDW and reticulocyte count, and reference standard was the responsiveness to a *therapeutic test with oral iron*. This report was made according to the requirements of the Standards for Reporting of Diagnostic Accuracy Initiative [28]. The validation studies on phase III are conducted with a population in whom it is clinically sensible to suspect the target disease in a pragmatic real-world setting of routine clinical practice [27]. Thereby, this study was conducted at a single pre-natal care center and compared the diagnostic tests in pregnant women previously classified as anemic, as they are under higher risk of iron-deficiency.

This study followed the ethics principles in research with human beings of the World Medical Association’s Declaration of Helsinki, and was approved by the Research Ethics Committee of *IMIP* (registration number 2050/10). Each participant was oriented, read and signed the consent form prior to their inclusion in the study. As we applied a before-after intervention with medicinal iron, our protocol was inscribed in International Clinical Trials Registry Platform (U1111-1123-2605) of WHO, and in Brazilian Registry of Clinical Trials (RBR-237 registered 28 July 2011, available at: http://www.ensaiosclinicos.gov.br/rg/RBR-237wbg).

### Participants and follow-up

Pregnant women attended at the prenatal care center of *Instituto de Medicina Integral Prof Fernando Figueira (IMIP)* were consecutively recruited based on a Hb value < 11 g/dL (WHO criteria for anaemia) [5] in the routinely prenatal exams plus the following inclusion criteria: 18 to 35 years old, 12^th^ to 32^nd^ weeks of low-risk singleton pregnancy. Our prenatal service composes of the reference network assistance for pregnant women in the public health system in the state of Pernambuco, in the Northeast of Brazil, and performs an average of 17,000 outpatient consultations and 6,000 deliveries per year. The exclusion criteria were Hb ≤ 7.0 g/dL, history of hypersensitivity or intolerance to ferrous sulfate, disorders or mental deficits; use of tobacco, alcohol or other drugs; previous diagnosis of another cause of anaemia; or infection at the time of the enrollment (positive serology for Human Immunodeficiency Virus or syphilis, leukocytosis or leukocyturia with positive urine culture, clinical signs and symptoms of airway infection).

Between August 2011 and October 2012, 187 women were consecutively enrolled and evaluated monthly for a period of 23 to 125 days, hence until December 2012, 139 women completed the trial. Sociodemographic information (age, self-assigned ethnicity, county of residence, educational level, family income *per capita*) and clinical-obstetric data (body mass index-BMI, numbers of pregnancies and births, time since the last delivery, weeks of current pregnancy and recent use of oral iron) were obtained at the time of the enrollment, and after the women were forwarded to blood collection to measure erythrocyte indices and serum ferritin pre-treatment (index-tests).

Every month, in addition to the routine of prenatal care, the participants responded a questionnaire about adverse effects (anorexia, heartburn, nausea, vomiting, diarrhea, constipation, colic or abdominal pain) and adherence to therapy, while the iron pills not taken were counted; and after they were forwarded to a new blood collection to measure the Hb post-treatment (follow-up of responsiveness to iron-therapy). The withdrawal occurred in case of high-risk pregnancy, genital bleeding, drop out of treatment or intolerance, and use of other iron supplements. The follow-up was stopped in case of childbirth, and cure (Hb ≥ 11.0 g/dL) or worsening of anaemia (Hb < 7.0 g/dL or drop in Hb > 1.0 g/dL). For more details, the protocol was previously published under the acronym AMA (it can be seen in supplementary material of this article - Additional file 1) [29].

### Test methods

The index-tests (erythrocyte indices and serum ferritin) and the reference standard test (*therapeutic test with oral iron*) were prospectively performed in all participants. The *therapeutic test with oral iron* began within the first 24 h after the blood collection for index-tests measures and consisted of two pills per day of ferrous sulfate with 40 mg of elemental iron (an algorithm of procedures and follow-up of the study can be seen in supplementary material of this article - Additional file 2).

The responsiveness to the *therapeutic test with oral iron* was measured in Z-scores of Hb, according to the method proposed by Beaton and McCabe [26] to normalize the haemodilution phenomenon. Each observed Hb value was converted, subject by subject, into standard deviation (SD) from the reference mean expected for the gestational week at the moment of the blood sampling. We used reference means of Hb reported by Centers for Disease Control and Prevention (CDC) [19, 20], which are based on the distribution of values of Hb in iron-supplemented and well-nourished pregnant women from developed countries. The criteria to define a positive reference standard test was defined a priori based on a theoretic *rationale* [29], as empirical data are rare. Anaemia was classified as iron-responsive by an Hb Z-score increase of at least 0.55 SD at the end of the intervention (difference between post and pre-treatment Hb Z-scores). This way, the reference standard results were obtained 23 to 125 days after the determination of the index-tests, which assured the blinding and the independence between them. The *rationale* used in developing our criteria on reference standard is described in details in our published protocol [29].

The erythrograms were analyzed by using the flow cytometry and absorbance with the automated hematology analyzer ABX Pentra DF120 manufactured by Horiba® and complemented by microscopic reading of smear stained with panoptic dye. This automated analyzer performs automatic calibration, validation and standardization for each sample tested. The reticulocyte counts were performed manually through the reading of blood smear stained with brilliant cresyl blue dye. The serum ferritin levels were obtained using the chemiluminescence immunoassay method with ADVIA equipment Centaur Ferritin, manufactured by Bayer®, whose detection limits are from 1 to 1650 ng/mL and the reference interval is of 10 to 291 ng/mL. The calibration of this immune assay is carried out according to the 2^nd^ international standard of WHO (WHO 80/578) [30].

The blood samples were collected and processed by the routinely procedures of the laboratory service of *IMIP*, which have governmental certification and follow standardized operational norms. The accuracy analysis was performed by applying the cutoff points of WHO for the 2^nd^ trimester of pregnancy [3], which correspond to two SD below the mean from the CDC [19, 20] reference population: red blood cell count < 3.8•10^12^cells/L; Hb < 10.5 g/dL; HTC < 32.0 %; MCV < 81.0 fL; MCH < 26.0 pg; MCHC < 32.0 g/dL; RDW > 14.0 %; reticulocyte count < 1.0 %; serum ferritin < 12.0 ng/mL.

### Sample size and data analysis

The sample size was calculated accounting for a sensitivity of 90 %, a specificity of 80 % and a maximum relative error of 10 %, resulting in a sample size comprised of 43 cases (iron-responsive anaemia) and 97 non-cases (not iron-responsive anaemia); but after the end of the study we had 37 cases plus 102 non-cases due to five exclusions *a posteriori*.

The data were double entered and validated using the Epi Info 3.5.4, and were analyzed with the software Stata/SE 12.1 and Open Epi 2.3.1. A dose-response effect of each iron pill ingested on the Hb Z-scores was verified in a linear regression model adjusted for days of treatment, laboratorial characteristics (initial values of Hb and serum ferritin) and other studied co-variables (sociodemographic, clinical-obstetric and therapeutic). These study population characteristics were compared between the two groups of cases and non-cases, using the Student’s t-test, Pearson’s Chi-square, Mann-Whitney test and Fisher’s exact test, considering a significance level of 5 %.

The diagnostic accuracy of erythrocyte indices and serum ferritin was estimated by calculating the sensitivity (Se), specificity (Sp), positive predictive value (PPV) and negative (NPV), positive likelihood ratio (LR+) and negative (LR-), diagnostic *Odds Ratio* (*OR*), area under the ROC curve (AUC), accuracy ratio and Cohen’s *kappa* coefficient (inter-rater agreement), and their respective confidence intervals of 95 %.

Briefly, the likelihood ratio is a probability quotient of a positive (LR+) or a negative (LR-) result between individuals with and without the target disease; so, the higher LR+ (over 1.0) more informative is a positive result for presence of the disease, while the lower LR- (under 1.0) more informative is a negative result for absence of the disease. The diagnostic *OR* is a ratio between both LR+ and LR-, so the higher *OR* higher the overall power of the test to discriminate the presence from the absence of disease [31]. On the other hand, the Cohen’s *kappa* coefficient is the proportion of agreements between index-test and reference standard, discounting the probability of random agreements, so the higher this coefficient more accurate is the index-test in relation to a reference standard; however, if both index and reference tests are inaccurate this coefficient can be high but clinically irrelevant [32].

**Fig. 1:**
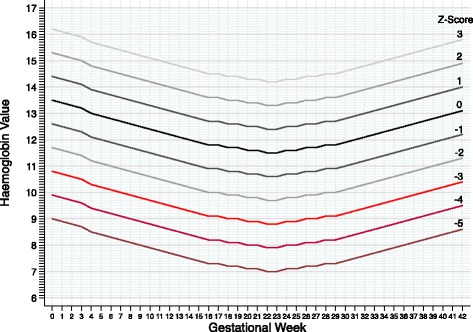
Nomogram with theoretical Z-scores curves of haemoglobin (Hb) in relation to the mean curve through gestational weeks from a reference population reported by Centers for Disease Control and Prevention [19, 20]

Those pregnant women who did not ingest the total doses of at least 1200 mg of elemental iron were excluded from these analyzes. In order to parameterize the asymmetrical distribution of the serum ferritin values in the sample, a logarithmic transformation it was performed; however, the ROC curve with log-transformed values did not differ from that with absolute values. Finally, as a study product, we have built a nomogram with hypothetical curves of negative and positive units of Z-scores in relation to the U-shaped curve of reference means of Hb by CDC at each gestational week (Fig. 1- *Nomogram of Hb Z-scores*) [19, 20].

## Results

### Study population characteristics

613 pregnant women with anaemia were identified at the *IMIP* laboratory web system and 487 were contacted by the research team and sought us at the time of their routine prenatal consultation. Among them, 246 met the inclusion criteria, but 24 did not accept to participate in the study and 35 were excluded, totaling a sample of 187 pregnant women, who were included and followed between August 2011 and December 2012 (a flow diagram showing inclusions, losses and failures to undergo index-testes and reference-standard test can be seen in the supplementary material of this article - Additional file 3). There were 25.7 % of losses: 43 follow-up losses and five exclusions for not taking the minimum effective iron doses (indeterminate results of the reference standard - Table 2), totaling 37 iron-responsive plus 102 not iron-responsive women. Women had ingested 31 to 180 iron pills during 23 to 125 days. This period differed of study protocol due to delays between monthly evaluations as they happened at the routine prenatal care.

The characteristics of the participants are shown in Table 1, according to the presence or not of responsiveness to the *therapeutic test with oral iron* (reference standard). Almost all of the pregnant women lived in the urban area. The sociodemographic and clinical-obstetric characteristics did not differ and the women using daily iron supplements for more than 1 month before the study (n 23) were homogeneously distributed between the groups.

**Table 1:** Comparison of sociodemographic, clinical-obstetric, laboratorial and therapeutic characteristics between iron-responsive and not iron-responsive pregnant women

	Classification		*P*
	Iron-responsive	Not iron-responsive	
	(n 37)	(n 102)^a^	
Sociodemographic, clinical-obstetric, laboratorial characteristics at enrollment			
Age (Mean ± SD)	26.3 ± 5.0	25.5 ± 4.7	0.387
Self-assigned ethnicity: n (%)			0.784
Pardo (brown)	23 (62.2)	54 (52.9)	
Black	6 (16.2)	20 (19.6)	
White	6 (16.2)	19 (18.6)	
Others	2 (5.4)	9 (8.8)	
BMI Atalah classification:^b^ n (%)			0.094
Underweight	6 (16.2)	21 (20.8)	
Health	24 (64.8)	47 (46.5)	
Overweight	2 (5.4)	22 (21.8)	
Obese	5 (13.5)	11 (10.9)	
Educational level: n (%)			0.255
Incomplete primary/middle school	1 (2.7)	4 (3.9)	
Complete primary/middle school	26 (70.3)	58 (56.9)	
Complete high school	10 (27.0)	32 (31.4)	
Complete graduation	0 (0.0)	8 (7.8)	
Family income *per capita* (*R$)*: Median (25^th^;75^th^centiles)	333.33 (269.58;483.33)	350.00 (203.33;524.75)	0.987
Months since the last delivery: Median (25^th^;75^th^centiles)	71.0 (44.3;90.6)	59.0 (44.8;85.0)	0.720
Nulliparous women: n (%)	21 (56.8)	58 (56.9)	0.991
Women using iron-supplement for ≥1 month: n (%)	3 (8.1)	20 (19.6)	0.083
Mean of Hb g/dL ± SD	10.1 ± 0.76	10.4 ± 0.58	0.018
Mean of Hb Z-scores ± SD	-1.8 ± 0.81	-1.4 ± 0.67	0.005
Serum ferritin (ng/mL): Median (25^th^;75^th^centiles)	19.0 (9.0;33.0)	28.7 (11.1;53.0)	0.060
Characteristics of the therapeutic test with oral iron			
Pregnancy weeks at start iron-therapy: Mean ± SD	21.6 ± 5.6	23.1 ± 4.6	0.121
Iron-therapy duration (days): Mean ± SD	52.6 ± 26.4	64.0 ± 26.1	0.025
Iron total doses: n (%)			0.046
1200–1760 mg	2 (5.4)	10 (9.8)	
1800–2400 mg	16 (43.2)	22 (21.6)	
>2400 mg	19 (51.4)	70 (68.6)	
Gastrointestinal symptoms after starting oral iron: n (%)	29 (78.4)	63 (61.8)	0.054

*Hb* haemoglobin concentration, *R$* current Brazilian coin, *SD* standard deviation of 95 %, *BMI* body mass index

^a^Among the not iron-responsive women, one woman had no data on income and another did not have the BMI

^b^Atalah *et al* 1997

The laboratory pre-treatment characteristics showed some differences between the two groups. The means of Hb and Hb Z-scores were significantly lower in the group with iron-responsive anaemia; and the median of serum ferritin levels tended to be lower in this group, without statistical significance. Regarding to the therapeutic characteristics, more than 90 % of the pregnant women in both groups ingested at least 1,800 mg of elemental iron until the follow-up end; the gastrointestinal complains were less frequent, while the duration and the number of iron pills ingested were higher among not iron-responsive women.

The erythrogram pre-treatment results were obtained from all participants, however, due to operational problems of the laboratory facility, 17 women did not have the reticulocyte count performed, occurring the same with serum ferritin in seven observations (lost results of index-tests or not performed in Table 2), which were excluded from each respective analysis. No participant reported adverse events by performing index-tests, such as local pain or bleeding due to blood sampling for erythrogram and serum ferritin measurements; but 66 % (92/139) of women complained of gastrointestinal symptoms after commencing the *therapeutic test with oral iron*.

**Table 2:** Index-tests (erythrogram and ferritin) and reference-standard (*therapeutic test with oral iron*) results (144 pregnant women)

	Response to the *therapeutic test with oral iron* (increase ≥ 0.55 in Hb Z-score)			
Index-tests	Present (iron-responsive)	Absent (not iron-responsive)	Total	Undefined
Red blood cell count				
Positive (<3.8•10^12^ cells/L)	32	79	111	04
Negative	05	23	28	01
Total	37	102	139	05
Haemoglobin				
Positive (<10.5 g/dL)	20	48	68	02
Negative	17	54	71	03
Total	37	102	139	05
HTC				
Positive (<32.0 %)	22	54	76	01
Negative	15	48	63	04
Total	37	102	139	05
MCV				
Positive (<81.0 fL)	07	14	21	01
Negative	30	88	118	04
Total	37	102	139	05
MCH				
Positive (<26.0 pg)	07	13	20	01
Negative	30	89	119	04
Total	37	102	139	05
MCHC				
Positive (<32.0 g/dL)	12	17	29	03
Negative	25	85	110	02
Total	37	102	139	05
RDW				
Positive (14.0 %)	08	25	33	00
Negative	29	77	106	05
Total	37	102	139	05
Reticulocyte count				
Positive (<1.0 %)	02	05	07	01
Lost or not performed	02	15	17	00
Negative	33	82	115	04
Total	37	102	139	05
Serum Ferritin				
Positive (<12.0 ng/mL)	12	25	37	01
Lost or not performed	02	05	07	00
Negative	23	72	95	04
Total	37	102	139	05

### Parameters of accuracy to predict the presence or absence of iron-responsive anaemia

Table 3 shows the accuracy parameters of erythrocyte indices and serum ferritin at the cutoff points recommended by WHO [3], observing that the PPV were around or below 40 %, while the NPV were all above 70 %. The erythrocyte mass indices (Hb and HTC) presented Se and Sp of around 50 %; the red blood cell count presented Se of 86 % with Sp of 22 %, while the reticulocyte count presented Se of 6 % with Sp of 94 %. The serum ferritin and all the erythrocyte morphology indices (MCV, MCH, MCHC and RDW) presented Se below 40 % and Sp above 70 %.

**Table 3:** Accuracy of erythrogram and ferritin to predict the responsiveness to iron-therapy in 139 pregnant women

Index-tests	Sensitivity	Specificity	Predictive Value +	Predictive Value -
	% (CI)	% (CI)	% (CI)	% (CI)
Red blood cell count (<3.8•10^12^cel/L)	86.5 (72.0;94.1)	22.5 (15.5;31.6)	28.8 (21.2;37.8)	82.1 (64.4;92.1)
HTC (<32.0 %)	59.5 (43.5;73.6)	47.1 (37.7;56.7)	28.9 (19.9;36.9)	76.2 (64.4;85.0)
Hb (<10.5 g/dL)	54.0 (38.4;68.9)	52.9 (43.3;68.9)	29.4 (19.9;41.1)	76.1 (64.9;84.5)
MCV (<81.0 fL)	18.9 (9.5;34.2)	86.3 (78.2;91.6)	33.3 (17.2;54.6)	74.6 (66.0;81.6)
MCH (<26.0 pg)	18.9 (9.5;34.2)	87.2 (79.4;92.4)	35.0 (18.1;56.7)	74.8 (66.3;81.7)
MCHC (<32.0 g/dL)	32.4 (19.6;48.5)	83.3 (74.9;89.3)	41.4 (25.5;59.3)	77.3 (68.6;84.1)
RDW	21.6 (11.4;37.2)	75.5 (66.3;82.8)	24.2 (12.8;41.0)	72.6 (63.5;80.2)
(>14.0 %)				
Reticulocyte (*n* = 122) (<1.0 %)	5.7 (1.58;18.6)	94.2 (87.2;97.5)	28.6 (8.2;64.1)	71.3 (62.4;78.8)
Serum ferritin (*n* = 132) (<12.0 ng/mL)	34.3 (20.8;50.8)	74.2 (64.7;81.9)	32.4 (19.6;48.5)	75.8 (66.3;83.3)

*CI* 95 % Confidence Interval, *HTC* (haematocrit), *Hb* (haemoglobin concentration), *MCV* (mean corpuscular volume), *MCH* (mean corpuscular haemoglobin), *MCHC* (mean corpuscular haemoglobin concentration), *RDW* (red blood cells distribution width)

### Parameters of accuracy to discriminate between presence and absence of iron-responsive anaemia

The LR + and LR- presented values very close to 1.0. The LR+ and LR- were just as close, resulting in diagnostic *OR* (ratio LR+ : LR-) slightly above or below 1.0. The AUC of serum ferritin was 0.61 and of the erythrocyte indices ranged from 0.51 to 0.64 (Table 4). All index-tests showed values of diagnostic accuracy (percentage of true tests) between 39.6 % (for red blood cell count; 95 % CI 31.8 to 47.9) and 69.8 % (for MCHC; 95%CI 61.7 to 76.8). In general, there were low rates of agreement between the index-tests and the reference standard, with the following values of the Cohen’s *kappa* test: red blood cell count = 0.05 (95 % CI -0.04 to 0.15); HTC = 0.05 (95 % CI -0.09 to 0.19); Hb = 0.05 (95 % CI -0.09 to 0.20); MCV = 0.06 (95 % CI -0.10 to 0.22); MCH = 0.07 (95 % CI -0.08 to 0.23); MCHC = 0.17 (95 % CI 0.00 to 0.33); RDW = -0.03 (95 % CI -0.19 to 0.14); reticulocyte count = 0.00 (95 % CI -0.12 to 0.12); serum ferritin = 0.08 (95 % CI -0.09 to 0.25).

**Table 4:** Accuracy of erythrogram and ferritin to discriminate iron-responsive and not iron-responsive pregnant women (n 139)

Index-tests	LR +	LR –	Diagnostic *Odds*	AUC
	Ratio (CI)	Ratio (CI)	Ratio (CI)	Area (CI)
Red blood cell count (<3.8•10^12^cel/L)	1.12 (1.08;1.16)	0.60 (0.30;1.19)	1.86 (0.65;5.33)	0.51 (0.40;0.61)
HTC (<32.0 %)	1.12 (1.02;1.24)	0.86 (0.72;1.03)	1.30 (0.61;2.80)	0.55 (0.44;0.66)
Hb (<10.5 g/dL)	1.15 (1.01;1.30)	0.87 (0.75;1.01)	1.15 (0.62;2.81)	0.58 (0.47;0.70)
MCV (<81.0 fL)	1.38 (0.36;5.26)	0.94 (0.88;1.01)	1.47 (0.54;3.98)	0.51 (0.40;0.62)
MCH (<26.0 pg)	1.48 (0.38;5.73)	0.93 (0.87;0.99)	1.60 (0.58;4.38)	0.57 (0.47; 0.68)
MCHC (<32.0 g/dL)	1.95 (1.23;3.07)	0.81 (0.75;0.88)	2.40 (1.01;5.69)	0.64 (0.54;0.74)
RDW (>14.0 %)	0.88 (0.34;2.32)	1.04 (0.96;1.12)	0.85 (0.34;2.10)	0.51 (0.41;0.62)
Reticulocyte (*n* = 122) (<1.0 %)	0.99 (0.20;4.89)	1.00 (0.91;1.10)	0.99 (0.18;5.38)	0.58 (0.47;0.68)
Serum ferritin (*n* = 132) (<12.0 ng/mL)	1.33 (0.90;1.97)	0.88 (0.80;1.97)	1.50 (0.65;3.46)	0.61 (0.50;0.71)

*LR* (Likelihood Ratio), *AUC* (Area Under Receiver Operating Characteristic Curve), *CI* 95 % (Confidence Interval), *HTC* (haematocrit), *Hb* (haemoglobin concentration), *MCV* (mean corpuscular volume), *MCH* (mean corpuscular haemoglobin), *MCHC* (mean corpuscular haemoglobin concentration), *RDW* (red blood cells distribution width)

### Therapeutic test with oral iron

There was a mean increase of 0.008 Hb Z-score for each iron pill (*p* = 0.013), independently of sociodemographic and clinical-obstetric characteristics, and pre-treatment values of serum ferritin and leucocytes, and adjusted for Hb pre-treatment and duration of treatment. This dose-response effect was not coincident with case criterion as we predefined, hence 30 pills of oral iron would result in an increase of 0.24 Hb Z-score rather than 0.55. For this reason, we estimated Se, Sp, PPV, NPV, LR and diagnostic *OR* also using cutoff points of 0.10, 0.20, 0.30 and 0.40 Hb Z-scores, but results were not sensitive to this criterion changes (see the multiple linear regression and accuracy reanalysis in supplementary material of this article - Additional files 4 and 5).

## Discussion

Our analysis showed that the serum ferritin (SF) levels and the values of the erythrocyte indices did not have a good performance to predict (Se, Sp and PV) and to discriminate (LR, diagnostic *OR*, accuracy, agreement and AUC) the iron-responsiveness among the anemic pregnant women studied. All index-tests presented PPV below 50 %, and the Hb and HTC (the most used erythrocyte indices) had both Se and Sp very low. The SF, morphological indices and young red blood cell (reticulocyte count) had low Se with high Sp; while the opposite occurred with the mature red blood cell count. The agreements between the results of any erythrocyte index or SF and the reference standard were *poor (kappa =* 0.00 to 0.19) [32]; and AUC were set in a *less accurate (0.5 < AUC ≤ 0.7)* range [33], so even their optimal cutoffs could not result in Se and Sp simultaneously above 50-60 %. The *post-test probabilities* of the disease (PPV) and non-disease (NPV) were close to the *pretest probabilities* (frequency) of cases (27 %) and non-cases (73 %), so a positive or negative result did not add diagnostics, as well as it was not an evidence *in favor (LR+ > 2.0)* or *against (LR- < 0.5)* the disease, resulting in *few informative (OR < 2.0)* tests [31].

Previous studies, which validated the erythrocyte indices against SF at 2^nd^ and 3^rd^ gestational trimesters, report similar low diagnostic performance with very low Se [14, 21, 22, 34]. Accordingly, in the National Health and Nutrition Examination Survey of United States (NHANES) 1999-2006, the Hb detected only 16 % (Se) of the pregnant women classified as iron-deficient by the ‘total body iron model’: {– [log_10_ (sTfR × 1000 ÷ ferritin) – 2.8229] ÷ 0.1207} < 0 mg/Kg [35]. ROC curves in a *moderately (0.7 < AUC ≤ 0.9)* or *less accurate* ranges [33] for both indices of erythrocyte mass and morphology were observed by Tam *et al* [10] (*versus* SF < 20 ng/mL), Casanova *et al* [9] (*versus* SF < 10 ng/mL) and Jaime-Pérez *et al* [14] (versus SF < 12 ng/mL). On the other hand, Tiwari *et al* [13] found a *highly accurate (0.9 < AUC < 1.0)* curve for Hb, with Se of 91 % and Sp of 79 % for a cutoff of 9.7 g/dL (*versus* SF < 12 ng/mL), differing from ours and other authors results.

The low diagnostic performance of SF in our study disagrees with studies comparing SF levels with the iron contents in the pregnant women’s bone marrow, which observed a positive correlation [11, 12]. Puolakka *et al* [12] found a Se of 72 % for SF < 35 ng/mL and Van den Broek *et al* [11] found a Se of 90 %, Sp of 85 % and LR+ of 6.0 for SF < 30 ng/mL, which was more accurate than serum iron, zinc protoporphyrin, transferrin saturation, transferrin iron-binding capacity and transferrin receptor. However, these small studies are not enough to validate the SF as a gold-standard for maternal iron-deficiency. The first one of these two studies occurred before international standardization of human ferritin for immune assays [12], and the other one included many women with HIV and Thalassemia [11].

Studies have shown gaps on standardization of diagnostic tests for maternal iron deficiency, precluding high level evidence [8]. Some differences between results found in previous validation studies and our own may be due to differences between the tests adopted as reference standard. While SF and bone marrow reflect the stored iron [3], the responsiveness to oral iron can reflect the physiologic balance between iron needs and intestinal absorption [36], and has been considered as a confirmatory test [3], even in pregnant women [7, 25]. Moreover, conventional criteria based on statistical cutoff points of erythrocyte indices and SF are static and do not seem coherent with the dynamic of body iron and plasma volume throughout pregnancy. However, despite of *therapeutic tests with oral iron* having already been applied in studies with children [37, 38] and with non-pregnant women [39], we were unable to find reports in pregnancy.

The reference standard was our main source of limitation, as patient compliance and physiologic changes of Hb (U-shaped curve of Hb) during pregnancy could bias the assessment of the *therapeutic test with oral iron* [26, 40]. From this perspective, gastrointestinal side-effects may influence whether women genuinely ingested iron pills, and may cause ascertainment-bias. To handle this issue, we monitored pill ingestion and registered adherence to an effective iron dose, as well as losses that would not exert an important effect on our analyses. In turn, the U-shaped curve of Hb, if not accounted for, could lead one to incorrectly conclude that physiological changes in Hb after a period of treatment were due to the oral iron [26, 40]. To correct this *curvilinear* phenomenon by including the gestational age (at start and end of iron-therapies) parametrically in a *linear* statistical model might not fit well [26]. On the other hand, a placebo group can be used for this purpose [1, 41, 42], but not at a real clinical setting.

For these reasons, we assessed the responsiveness to oral iron, subject by subject, by Hb Z-scores [19, 20], and then we adopted it as a dependent variable in a regression model to estimate a dose-response effect per iron pill. The adjusted coefficient of 0.008 Z-score per pill was robust and agreed with meta-analyzes adjusted for control groups, which estimate Hb increases of 0.4 g/dL in pregnant women, after a few weeks of low daily doses of oral iron [1, 42]. Two previous works have used Hb Z-scores to assess oral iron-therapies during pregnancy, but both of them aimed to compare intermittent with daily treatments, rather than to estimate a dose-response effect [26, 43]. The significant per pill effect in our analysis indicates that we could identify minimal therapeutic responses, within the broad period of iron-therapies in this trial (23–125 days), particularly because women ingested therapeutic doses [41] in both groups of iron-responsive and not iron-responsive anaemia.

Finally, in our study population, if the decision to treat was based on Hb or SF, half of women would undergo iron-therapy without hematological benefit or would not receive treatment despite having iron needs. This finding agrees with the estimate that only half of the global burden of anaemia can be attributed uniquely to iron-deficiency [4, 44], and that only half of anaemic pregnant women get cured after taking adequate amounts of iron in clinical trials [1, 41]. Actually, if the response to the *therapeutic test with oral iron* was taken into account, most of the pregnant women studied would be identified as not iron-responsive. This result may point towards the presence of other causes of anaemia and futile treatments, since these women were treated longer and with higher iron doses. Nevertheless, a lack of empirical data during pregnancy may bring doubts about how sensitive to the *therapeutic test with oral iron* would be women with severe anaemia, as well as if other biomarkers could be correlated with iron-responsiveness more than those we had selected.

## Conclusions

Erythrocyte indices and serum ferritin have low ability to predict and discriminate the iron needs in women with mild-moderate anaemia at 2^nd^ or 3^rd^ trimesters of pregnancy. On the other hand, a *therapeutic test with oral iron* in low doses may timely identify less iron-responsive cases. We recommend using *Nomograms of Hb Z-scores* as a practical and low-cost tool to normalize the haemodilution effect on Hb therapeutic changes during routinely prenatal care; but they should be built and validated for each country or world region, as well as new diagnostic tests for maternal iron-deficiency should be investigated.

## Abbreviations

AUC, area under the ROC curve; BMI, body mass index; CDC, Centers for Disease Control and Prevention; Hb, haemoglobin; HTC, haematocrit; *IMIP, (Instituto de Medicina Integral Prof Fernando Figueira)*; LR, negative likelihood ratio; LR+, positive likelihood ratio; MCH, mean corpuscular haemoglobin; MCHC, mean corpuscular haemoglobin concentration; MCV, mean corpuscular volume; NPV, negative predictive value; *OR, odds ratio*; PPV, positive predictive value; RDW, red blood cell distribution width; ROC, receiver operating characteristic; SD, standard deviation; Se, sensitivity; SF, serum ferritin; Sp, specificity; STARD, Standards for Reporting of Diagnostic Accuracy Initiative; WHO, World Health Organization

## Additional files

Additional file 1: Study protocol. (PDF 372 kb) Additional file 2: Algorithm of procedures and follow-up of the study. (PDF 142 kb) Additional file 3: Flow diagram showing inclusions, losses and failures to undergo index-testes and reference-standard test. (PDF 110 kb) Additional file 4: Regression model of dose-response effect of therapeutic test with oral iron (DOCX 14 kb) Additional file 5: Re-analyses of Se, Sp, PPV, NPV and OR using several cutoffs for the therapeutic test with oral iron (DOCX 22 kb) Additional file 6: Data set of AMA study (XLSX 64 kb)
