# Supplementary figures and images for: Could the erythrocyte indices or serum ferritin predict the therapeutic response to a trial with oral iron during pregnancy? Results from the Accuracy study for Maternal Anaemia diagnosis (AMA)
Source: BMC Pregnancy Childbirth. 2016 Aug 12;16:218. doi: 10.1186/s12884-016-1005-x (PMC4982235; doi:10.1186/s12884-016-1005-x)

# ALGORITHM OF INCLUSION AND FOLLOW-UP PROCEDURES OF WOMEN IN THE AMA STUDY

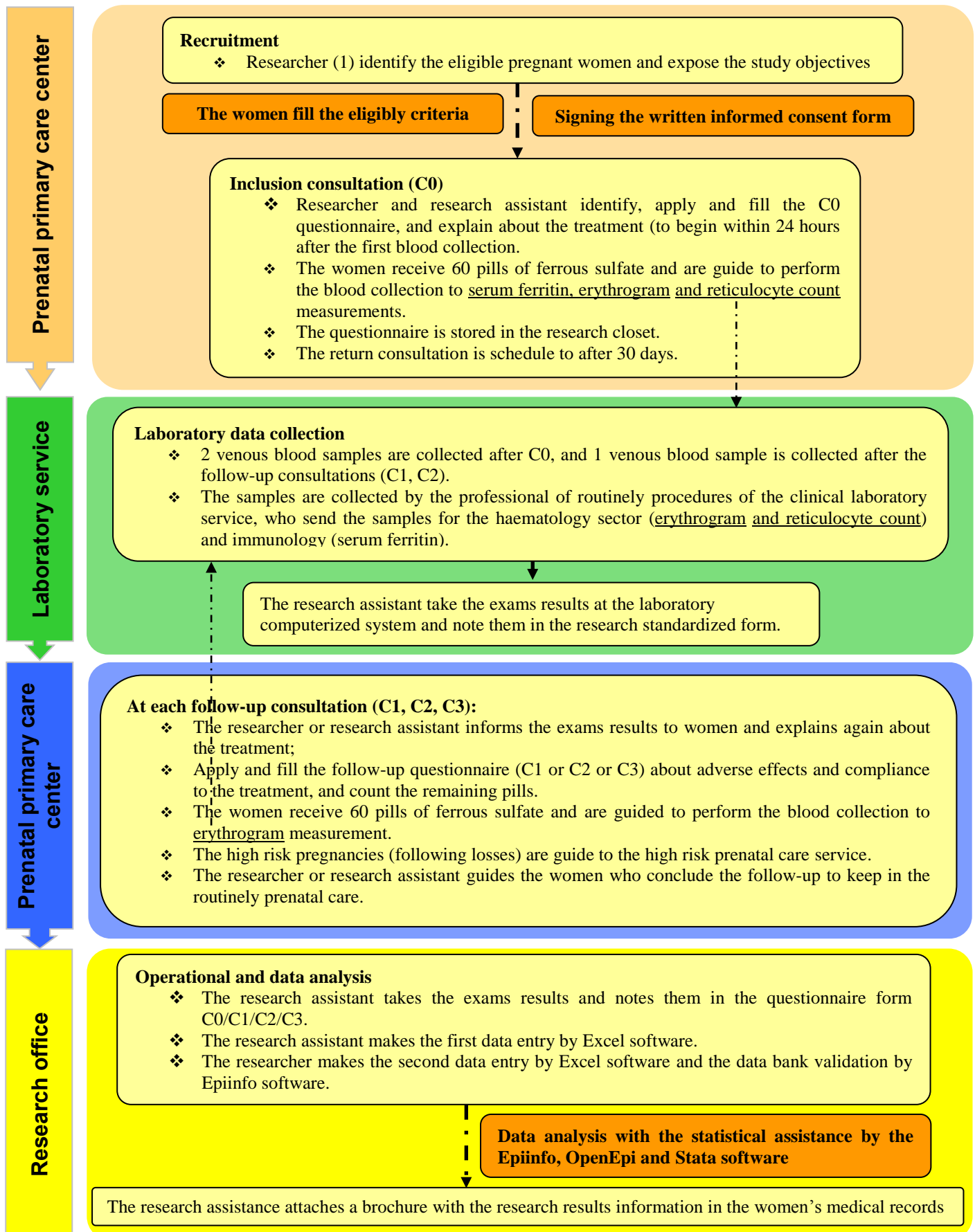

Supplement: Additional file 2: — Algorithm of procedures and follow-up of the study. (PDF 142 kb) [file 12884_2016_1005_MOESM2_ESM.pdf]
