# Supplementary material for: Could the erythrocyte indices or serum ferritin predict the therapeutic response to a trial with oral iron during pregnancy? Results from the Accuracy study for Maternal Anaemia diagnosis (AMA)
Source: BMC Pregnancy Childbirth. 2016 Aug 12;16:218. doi: 10.1186/s12884-016-1005-x (PMC4982235; doi:10.1186/s12884-016-1005-x)
Supplement: Additional file 3: — Flow diagram showing inclusions, losses and failures to undergo index-testes and reference-standard test. (PDF 110 kb) [file 12884_2016_1005_MOESM3_ESM.pdf]

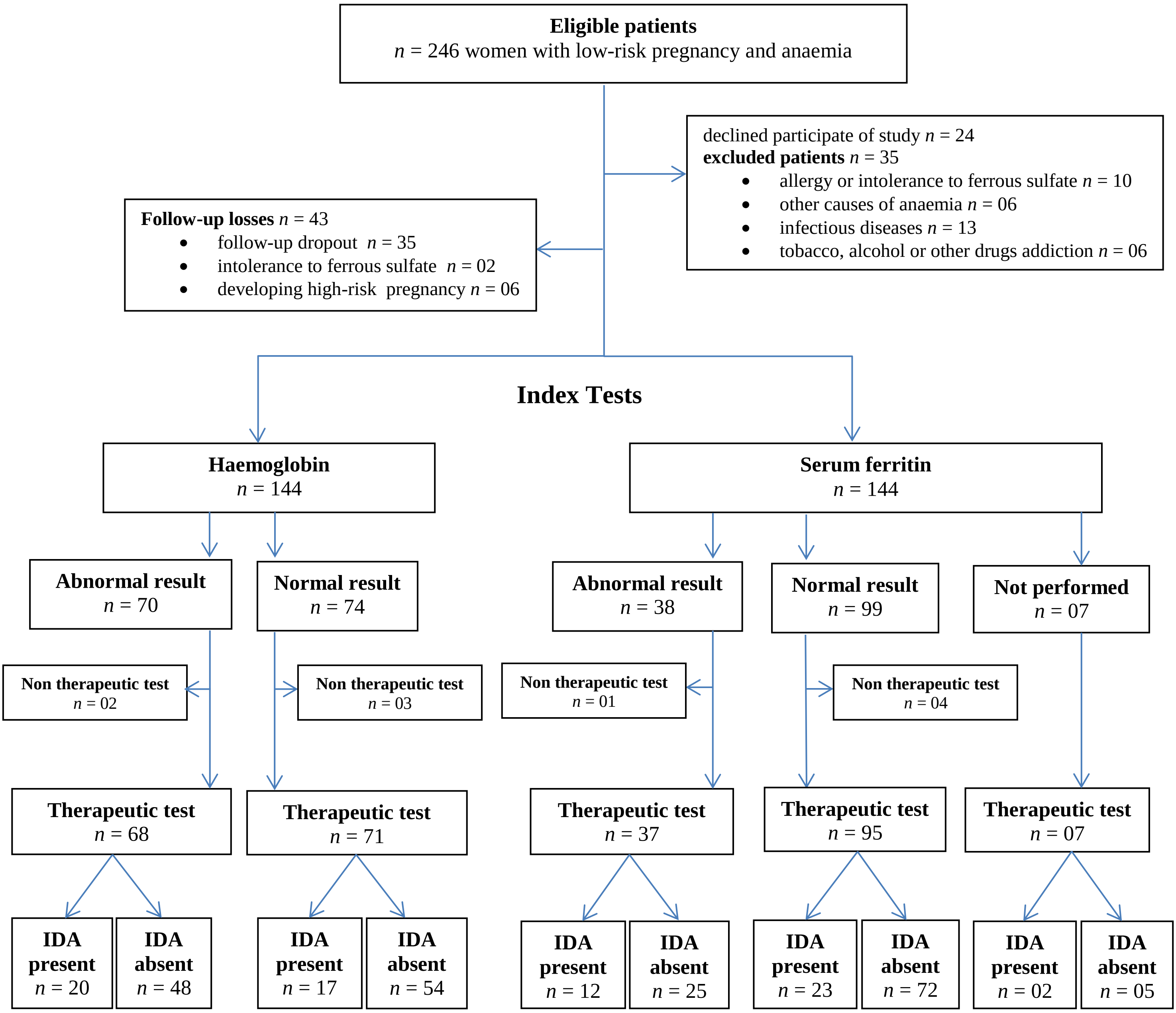

Flow diagram - inclusions, losses and failures to undergo index-tests (haemoglobin and serum ferritin) and reference standard test (*therapeutic test with oral iron*) for iron deficiency anaemia (IDA), among 144 pregnant women recruited and followed between August 2011 and December 2012.
