# Supplementary material for: Could the erythrocyte indices or serum ferritin predict the therapeutic response to a trial with oral iron during pregnancy? Results from the Accuracy study for Maternal Anaemia diagnosis (AMA)
Source: BMC Pregnancy Childbirth. 2016 Aug 12;16:218. doi: 10.1186/s12884-016-1005-x (PMC4982235; doi:10.1186/s12884-016-1005-x)
Supplement: Additional file 4: — Regression model of dose-response effect of therapeutic test with oral iron (DOCX 14 kb) [file 12884_2016_1005_MOESM4_ESM.docx]

Regression analysis of dose-response effect of each iron pill on post-pretreatment difference of Hb Z-scores; Brazilian pregnant women, 2012.

| **Variables** | **Hb Z-score Mean Difference (SD)** | | | | | | | | | | | |  |
| --- | --- | --- | --- | --- | --- | --- | --- | --- | --- | --- | --- | --- | --- |
| Iron pills | .008 (.003)**^**^** | | | | .007 (.003)****** | | | | .008 (.003)******* | | | |  |
| Days of treatment | -.014 (.006)*** | | | | -.014 (.006)****** | | | | -.018 (.006)******* | | | |  |
| **Adjusted for socio and obstetrics characteristics** (ethnicity, education, family income, parity, inter gestation interval) | |  | | **NO** | | | **YES** |  | | | **YES** |  | |
| **Adjusted for clinical characteristics**  (nutritional classification^†^, previous iron supplementation; baseline Hb, ferritin and leucocytes) | |  | | **NO** | | **NO** | | | | **YES** | |  | |
| Constant |  | |  | | .164 (.158) | | | | .417 (.410) | | | |  |
| R^2^ | .059 | | | | .073 | | | | .230 | | | |  |
| N. Obs. | 137 | | | | 137 | | | | 136^††^ | | | |  |

SD: Standard Deviation ⎜95% CI: Confidence Interval of 95%

*****p value < .10; ******p value < .05; *******p value < .01; **^†^**Atalah *et al* 1997.
