# Supplementary material for: Could the erythrocyte indices or serum ferritin predict the therapeutic response to a trial with oral iron during pregnancy? Results from the Accuracy study for Maternal Anaemia diagnosis (AMA)
Source: BMC Pregnancy Childbirth. 2016 Aug 12;16:218. doi: 10.1186/s12884-016-1005-x (PMC4982235; doi:10.1186/s12884-016-1005-x)
Supplement: Supplementary file 5 [file 12884_2016_1005_MOESM5_ESM.docx]

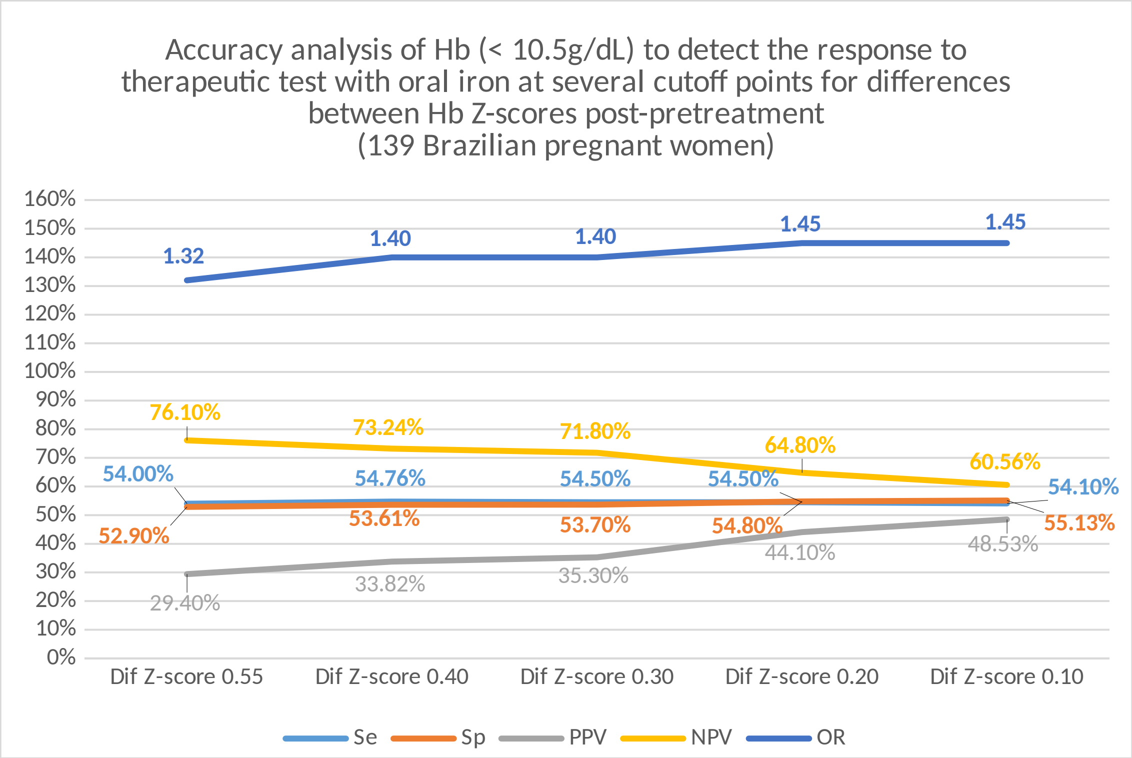
Accuracy analysis of Hb (< 10.5g/dL) to detect the response to therapeutic test with oral iron at several cutoff points for differences between Hb Z-scores post-pretreatment (139 Brazilian pregnant women)
160%
150%
140%
130%
120%
110%
100%
90%
80%
70%
60%
50%
40%
30%
20%
10%
0%
1.32
1.40
1.40
1.45
1.45
76.10%
73.24%
71.80%
64.80%
60.56%
54.00%
54.76%
54.50%
54.50%
54.10%
52.90%
53.61%
53.70%
54.80%
55.13%
29.40%
33.82%
35.30%
44.10%
48.53%
Dif Z-score 0.55
Dif Z-score 0.40
Dif Z-score 0.30
Dif Z-score 0.20
Dif Z-score 0.10
Se
Sp
PPV
NPV
OR

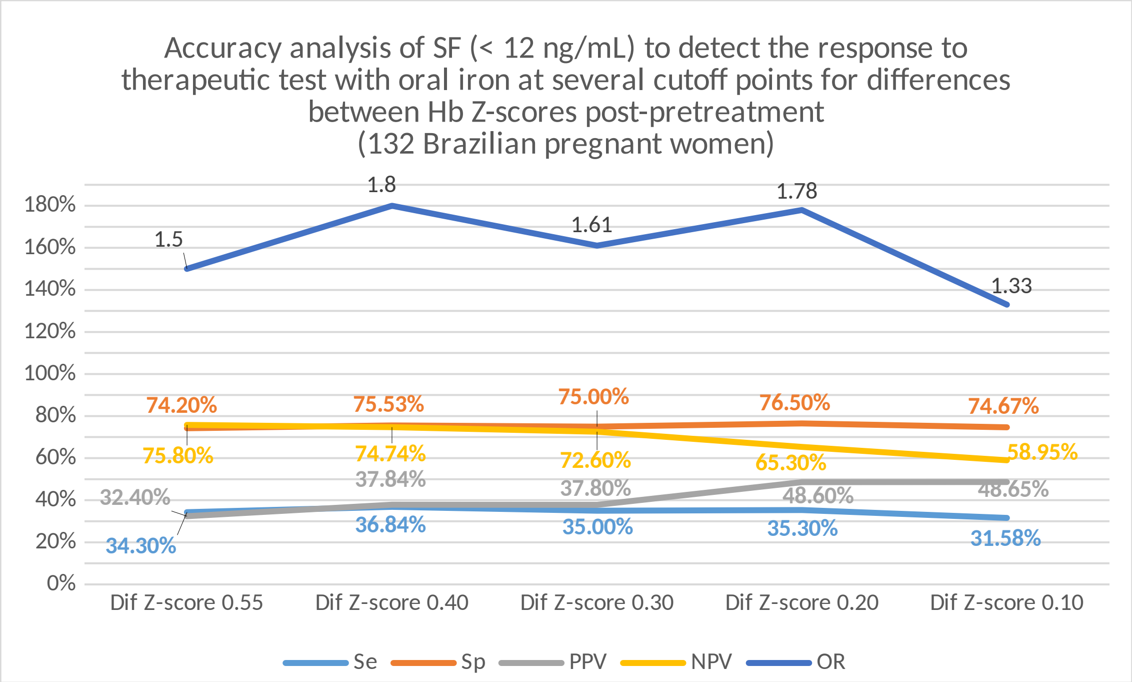
Accuracy analysis of SF (< 12 ng/mL) to detect the response to therapeutic test with oral iron at several cutoff points for differences between Hb Z-scores post-pretreatment (132 Brazilian pregnant women)
180%
160%
140%
120%
100%
80%
60%
40%
20%
0%
1.5
1.8
1.61
1.78
1.33
74.20%
75.53%
75.00%
76.50%
74.67%
75.80%
74.74%
72.60%
65.30%
58.95%
32.40%
37.84%
37.80%
48.60%
48.65%
34.30%
36.84%
35.00%
35.30%
31.58%
Dif Z-score 0.55
Dif Z-score 0.40
Dif Z-score 0.30
Dif Z-score 0.20
Dif Z-score 0.10
Se
Sp
PPV
NPV
OR
